# Melliferous insects and the uses assigned to their products in the northern Yungas of Salta, Argentina

**DOI:** 10.1186/s13002-018-0222-y

**Published:** 2018-04-11

**Authors:** Fabio Fernando Flores, Norma Inés Hilgert, Liliana Concepción Lupo

**Affiliations:** 1 Cátedra de Ecología General y Laboratorio de Palinología, Facultad de Ciencias Agrarias, Universidad Nacional de Jujuy, Alberdi 47, (4600) San Salvador de Jujuy, Jujuy Argentina; 2 Instituto de Biología Subtropical, IBS-CONICET, Universidad Nacional de Misiones, Facultad de Ciencias Forestales, Universidad Nacional de Misiones, Asoc. Civil CeIBA, Bertoni 85, (3370) Puerto Iguazú, Misiones Argentina; 3 Instituto de Ecorregiones Andinas (INECOA), Universidad Nacional de Jujuy – CONICET, Alberdi 47, (4600) San Salvador de Jujuy, Jujuy Argentina

**Keywords:** *Plebeia* sp. nov., Ethnozoology, Diversity, Medicinal uses, Nutraceutical medicine, Stingless bee, Baritú

## Abstract

**Background:**

The order Hymenoptera comprises melliferous insects (bees, wasps and bumblebees); among them, stingless bees comprise a diverse group of eusocial insects present in tropical and subtropical areas. Of a total of approximately 500 species, 400 are found in the Neotropics. On the continent of America, before the introduction of *Apis mellifera*, these insects represented the main source of honey and wax. In Argentina, ethnobiological investigations had been carried out on this group of insects, principally in the Atlantic Forest and Chaco regions. Out of a total of 33 species, only 14 were recorded for use or breeding. In the Yungas, however, there are no ethnobiological studies analyzing this group of species, although the use of their products is mentioned in different ethnobotanical works. This paper studies the knowledge and uses of melliferous insects by the inhabitants of the village of Baritú and surrounding.

**Method:**

Information on location, management and duties assigned (e.g., preparation and administration) to deal with bee products like honey, pollen, wax and propolis was obtained through semi-structured interviews. Besides, reference material was collected to identify melliferous insects known and used in the region.

**Results:**

Fifteen ethnospecies were identified and grouped locally according to their defensive behavior. The culturally most important species is the stingless bee *Plebeia* sp. nov.—*mansita-*, in terms of frequency of citations and diversity of uses, and among those that sting, the honeybee *Apis mellifera*—*extranjera-*. Honey, pollen, wax, and propolis of *Plebeia* sp. nov. had the highest current frequency of use. Honey is used in food (incorporated at pure state, as a complement and in drinks), as nutraceutical food and in medicinal preparations. In addition, it is an important resource for marketing during the warm season, infusions being the main mode of administration. Pollen is used as a supplement for food and alcoholic drinks, wax mainly in candle making, and propolis.

**Conclusion:**

The data obtained in this study complements the information available in ethnobotanical studies carried out in the region. The present study is the first on melliferous insects in the area. A new species of stingless bee the genus *Plebeia* was registered, and it was observed that the known distribution of others has increased.

## Background

The order Hymenoptera comprises melliferous insects (bees, wasps and bumblebees); among them, stingless bees comprise a diverse group of eusocial insects present in tropical and subtropical areas. Of a total of approximately 500 species, 400 are found in the Neotropics [1]. Before the introduction of *Apis mellifera* L., stingless bees represented the main source of honey and wax in the tropical and subtropical American continent [2–11].

Usually, only part of the species known in a certain region is of interest to humans, and practically, all products of hives are exploited indicating the importance of this resource for the inhabitants of the regions, as was recorded, for example in Brazil [2, 12], Colombia [13], Costa Rica [14], Mexico [5, 15], Peru [9, 15], and Argentina [16].

In Argentina, ethnobiological studies over the last few years have seen considerable progress, especially in the Atlantic Forest [11, 16–19] and the Chaco regions [20–23]. Out of a total of 33 species, only 14 were recorded for use or breeding [24]. In the Yungas, the use of their products is mentioned in different ethnobotanical works. In them, the inhabitants of the region refer to bees and their honey indicating the importance of these insects and their products for barter as well as for domestic medicine [25, 26].

Ethnobiology provides an excellent, theoretical and conceptual framework for the study of honeys since it integrates the analyses of cultures, their values and their environments [27]. Within the discipline, animal and vegetable resources, as well as medicinal uses of edible products, are frequently analyzed separately. In this paper, we discuss the uses of honey as food or medicine and analyze their functional value. For example, when honey is consumed in order to strengthen the organism, we consider it as functional food, contemplating the overlap between food and medicine proposed in previous studies [28–31].

The general objective of this work is to highlight the list of melliferous species that are used and the uses assigned to their products (honey, pollen, wax) as well as the way of locating and managing hives in the village of Baritú and surroundings areas bordering the Baritú National Park in the north of the Argentine Yungas.

## Methods

### Study area

**Fig. 1:**
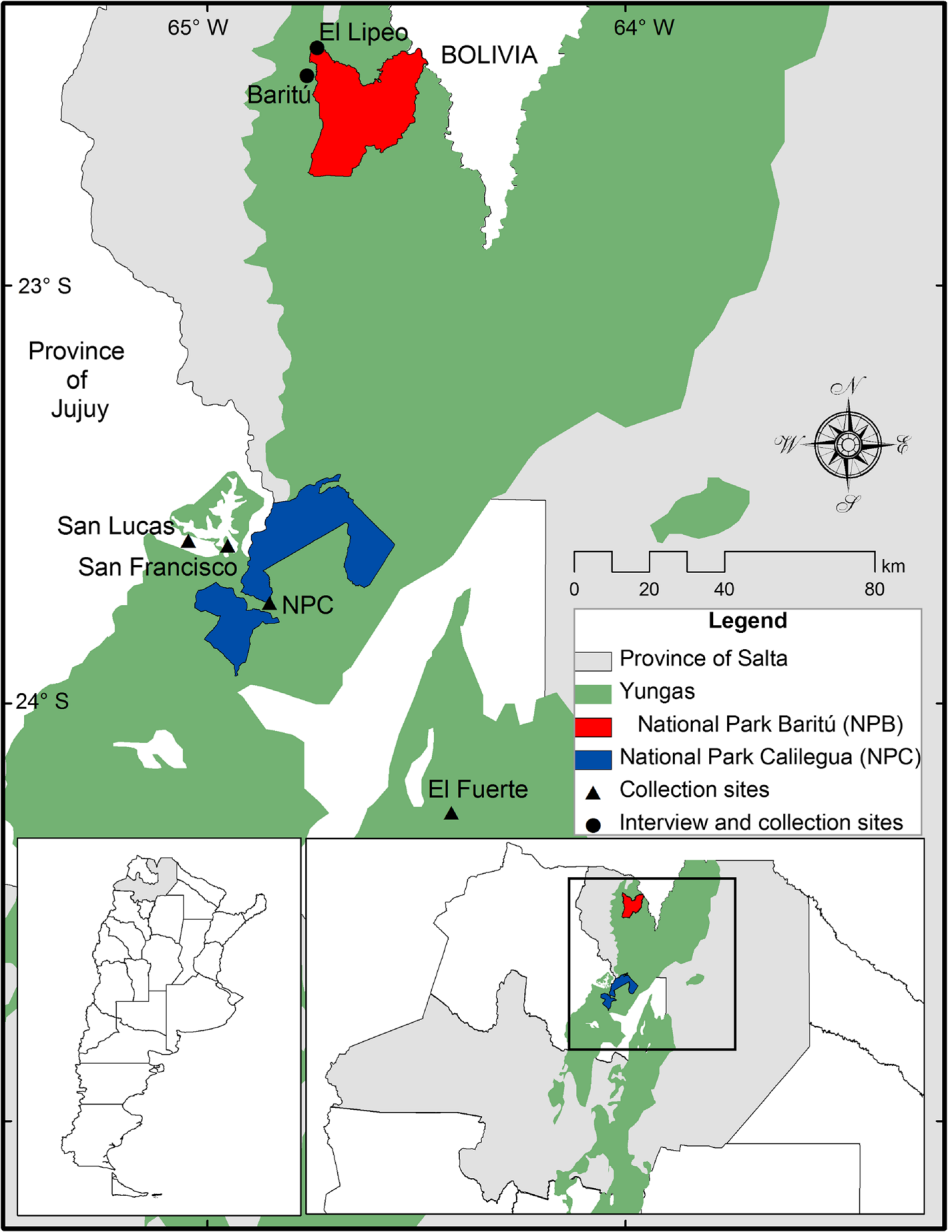
Location of the study area next to Parque Nacional Baritú within the Yungas phytogeographic province

Our study was carried out in the village settlements of Baritú and surroundings, located in the department of Santa Victoria, in the province of Salta (22° 28' 54.1'' S, 64° 45' 39.4" W). The area is 45 km from Los Toldos and 16 km from the community of El Lipeo. Its inhabitants are dispersed along small streams; houses and nearby crop areas are located between 1546 and 1700 masl (Fig. 1).

The climate is subtropical with a dry season during the months of April to November, with mild and very humid summers and cold and also humid winters without rainfall. The average annual temperature is 21 °C, with a minimum of − 7 °C and a maximum of 39 °C. As in all cloud forests, two forms of precipitation are observed: rainfall reaches approximately 3000 mm per year and is especially concentrated from November to April and precipitation by condensation—horizontal precipitation—is habitual during the dry season. From the phytogeographic point of view, the area is located in the Yungas and local vegetation represents a transition of the different ecological layers of the Submontane Subtropical Forest and the Montane Mosit Forest [32, 33].

### Sociocultural characteristics

The present population consists of members who are descendants of native Andean Amerindians and Spanish colonists; they speak Spanish with some Quechua words [25]. At present, the Lipeo and Baritú Aboriginal Communities belong to the “Upper basin of the Lipeo river” Indigenous Community, formed by the communities of Baritú, El Lipeo and El Arazay (http://barituparquenacional.blogspot.com.ar/p/comunidades-indigenas.html) (as from July 30, 2015).

According to health post records, the number of families living in the village and neighboring setllements (Abra de Minas and El Lipeo) amounts to 45, with a total of 163 people (91 men and 72 women), 55% of whom are more than 20 years old. However, the population of the region is very dynamic according to the time of year (for example, in 2013, 143 people were registered in the same records). These variations are mainly due to young adults who travel for seasonal work (to the wine harvest in the provinces of Mendoza and San Juan, the citrus harvest in other localities in the provinces of Salta, Jujuy and Tucumán, etc.) and/or emigrate to urban centers to complete their academic training (tertiary or university level) (Baritú Health Post, unpublished material). In terms of institutions, Baritú has a health post and a primary multigrade school.

Families live in a subsistence economy, with products destined mainly for self-consumption. The main productive activities are shifting agriculture and livestock migration (called *transhumance*) in agreement with other sectors of the Upper Basin of the Bermejo River [32, 34, 35]. The main crops are corn—*maíz—*(*Zea mays* L.) and potatoes—*papas*—(*Solanum tuberosum* L.), together with peanuts—*maní*—(*Arachis hipogaea* L.), peppers—*ajíes*—(*Capsicum* sp.), black-seed squash—*cayotes—*(*Cucurbita ficifolia* Bouché) and pumpkins*—zapallos—*(*C. maxima* Duchesne), among others [36]. In the area, agriculture is developed in permanent plots—corrals or pastures—bordering the dwellings and in dismantled sites of temporary use*—chacras*—[36]. Additional family income comes from temporary work, handicrafts [37], and/or pensions and social plans.

### Food and domestic medicine in the region

In previous studies, we recorded 242 vegetable ethnospecies (folk names given to one or several species) used for medicinal purposes by the population of the region and its surrounding area, including those used in health centers and in formal medicine [25, 38]. Medicinal practices present elements shared with towns in the Puna and Prepuna, probably as a remnant of the ancient influence of the Kallawayas-itinerant herbal physicians who traveled the region from different parts of Bolivia [39–41]. The balance between health and illness can be lost by supernatural causes (*susto*, *aire*, etc.), cultural causes (breaking rules or social taboos), and natural causes (such as an imbalance between the individual and his environment) [39, 41].

### Data collection and analysis

To select collaborators, randomly open interviews were initially conducted in order to establish a trust relationship with the villagers and to identify the key local interlocutors. We registered the terms used to refer to melliferous insects, their hives, and the uses of their products. The incorporation of the semantic domain related to the topic facilitated the inquiries and discussions with the settlers [42–44]. In the article, these expressions are cited in *italics* and their meanings are indicated the first time they appear coined.

At the beginning of the activities, oral informed consent was obtained from all those involved in the consultations and visits, as recommended by the code of ethics of the International Society of Ethnobiology [45]. Between September 2011 and August 2013, visits were made, together with specialists and/or regular users, to the sites where they habitually search for these resources. The area is locally known as *el monte* (which refers to forest sectors, with good to regular conservation status, situated generally in warm and distant lands located lower than those of the village, about 3–6 h of pedestrian road from it). Whenever possible, searches of wild nests and collections of its used products were carried out during such visits (although most of the hives were not harvested in our presence).

Ethnobiological walks were performed to collect samples of the used melliferous insects and to know which of their resources were used. In the dwellings, semi-structured interviews were carried out on known and used melliferous ethnospecies, the uses assigned to their products (e.g., honey, pollen, wax, propolis) and the way they were prepared and administered (sensu Bernard [42]; Martin [44] and Guber [46]).

The information was recorded with a digital voice recorder, and annotations were made in field notebooks. Twenty-nine people were included in the study (15 men aged between 18 and 66, and 14 women aged between 20 and 76). Each hive located was photographed, and a descriptive record of the observable characteristics of the ethnospecies and their nest was made. Samples of each ethnospecies were collected and deposited in two entomological collections, at the Museo de La Plata and in the collection of the Instituto de Biología Subtropical. The identification of the species was made by Dr. Leopoldo J. Álvarez from the Museo de La Plata—División Entomología—Universidad Nacional de La Plata. For the collection of reference material, the corresponding permits were obtained from the Administración de Parques Nacionales (Project Permit No. 05/2011, No.: 28/2012 and Renewal 1/2013). Herbarium specimens from the plants referred to in applications were also collected and deposited in the herbarium JUA of the Universidad Nacional de Jujuy. For botanical nomenclature, we followed the criteria of The Plant List (http://www.theplantlist.org/).

For non-collected ethnospecies, possible identities are suggested taking into account the physical and behavioral descriptions provided by locals, the potential area of distribution of Meliponini species [24, 47], and the information on vernacular uses and names in neighboring biomes available in the literature [20, 48]. Finally, for the conceptual organization regarding ethnospecies, the latter were grouped into those that sting and do not sting (Table 1) following the categorization made by Zamudio [11]. The collected data were analyzed qualitatively.

**Table 1:** Ethnospecies mentioned by the inhabitants of Baritú and in others sectors of the Argentine Yungas

	Baritú and neighboring villages (Department of Santa Victoria, province of Salta)				Yungas of province of Jujuy
	Clasification	Vernacular name (VN)	Family and tribe taxonomic	Scientific name	
Ethnospecies that do not sting	Stingless bees	*brava, bravita* (*)	Apidae, Meliponini	*Scaptotrigona jujuyensis* (Schrottky)	Locality of San Francisco (department Valle Grande), collection date: 07/28/2010, VN: *negrillo.*
		*cidra, igra* (*)	Apidae, Meliponini	*Paratrigona glabella* Camargo & Moure.	*–*
		*cherlinca* (*)	Apidae, Meliponini	*Plebeia catamarcensis* (Holmberg)	*–*
		*la burra*, *la burra toronjila* (*)	Apidae, Meliponini	*Lestrimelitta rufipes* (Friese)	Calilegua National Park (department Ledesma), Collection date: 05/08/2012. In this explored sector, we have data of the presence of the genus *Lestrimelitta* that probably correspond to the same species of melipona cited in this study.
		*mansita*	Apidae, Meliponini	*Plebeia* sp. nov.	Locality of San Lucas (department Valle Grande). Collection date: 11/16/2010, VN: *negrillo.* Collection date: 12/16/2010, VN: *arropillo.*
		*moro moro* (¥)	Apidae, Meliponini	Cfr. *Melipona*	–
		*posquillo* (*)	Apidae, Meliponini	Cfr. *Plebeia doryana* (Friese)	Locality of San Lucas (department Valle Grande), collection date: 1/6/2010. Locality El Fuerte (department Santa Bárbara), collection date: 1/0/2010. VN *pusquillo*
		*señorita* (*)	Apidae, Meliponini	*Tetragonisca fiebrigi* (Schwarz).	Calilegua National Park (department Ledesma), collection date: 09/13/2011, VN: *mestizo*
		*yana* (¥)	Apidae, Meliponini	Cfr. *Scaptotrigona*	–
Ethnospecies that sting	Wasp	*carnicero*	Vespidae, Epiponini	*Agelaia pallipes* (Olivier)	*–*
		*chillaguata*, *poronguillo* (*)	Vespidae, Epiponini	*Polybia ruficeps* (Schrottky)	*–*
		*chesgua rubia grande*	Cfr. Vespidae	–	–
	Honeybee	*extranjera*	Apidae, Apini	*Apis mellifera* Linnaeus.	*–*
	Bumblebee	*guancoiro*, *guanquero*	Apidae, Bombini	*Bombus atratus* Franklin, H. J.	*–*
		*paraguaya*	Apidae, Bombini	Cfr. *Bombus*	–

(*)Ethnospecies present in El Lipeo or from warmer locations mentioned by people born in El Lipeo and living now in Baritú

(¥)Ethnospecies of lower zones from the Pre-montane forest of Yungas or the Chaquenian phytogeographic area, mentioned by people from other locations within these phytogeographic characteristics

## Results

### Useful melliferous insects

**Fig. 2:**
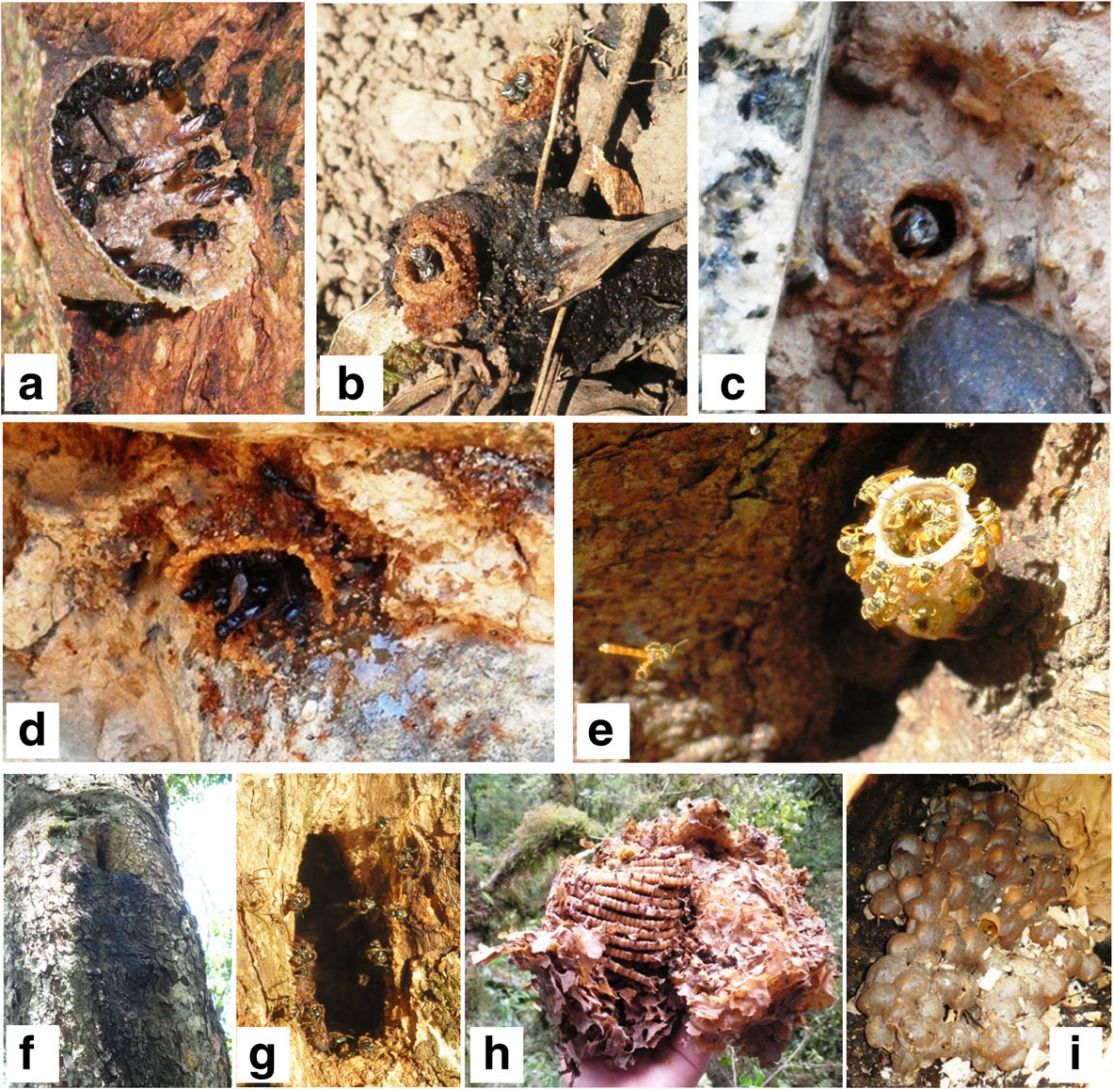
Ethnospecies that do not sting. **a** *brava, bravita* (*Scaptotrigona jujuyensis*); **b** *cidra, igra (Paratrigona glabella)*; **c** *cherlinca* (*Plebeia catamarcensis*); **d** *la burra, la burra toronjila* (*Lestrimellita rufipes*); **e** *señorita* (*Tetragonisca fiebrigi*); **f**–**i** *mansita* (*Plebeia* sp. nov.). **f** Access to the nest in a trunk of *palo yerba, yerba blanco* (*Ilex argentina* Lillo, Aquifoliaceae). **g** Individuals in the entrance of a nest. **h** Nest with the presence of involucro (wax sheets) around it. **i** Honey pots

**Fig. 3:**
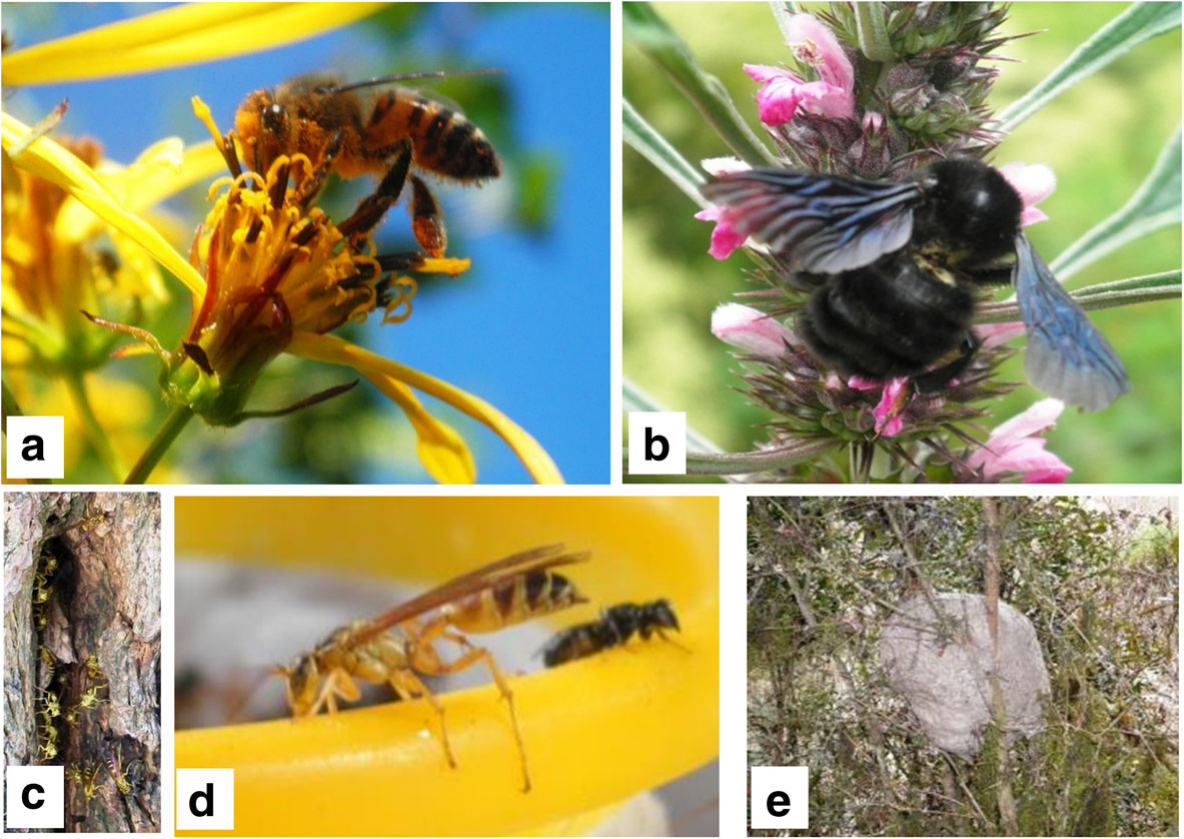
Ethnospecies that sting. **a** *extranjera* (*Apis mellifera*); **b** *guanquero, guancoiro* (*Bombus atratus*); **c**, **d** *carnicero* (*Agelaia pallipes*). **e** Hive of *chillaguata, poronguillo* (*Polybia ruficeps*) hanging from a branch of *pino del cerro* (*Podocarpus parlatorei* Pilg., Podocarpaceae)

During the study, we recorded 15 melliferous ethnospecies known to locals, belonging to Apidae (12) and Vespidae (3) families. Until now, 9 genera and 10 species have been identified (Table 1). Figures 2 and 3 show images of the collected ethnospecies. The ethnospecies richness cited per interviewee ranged from 2 to 11, a mode of 4 and an average of 6. According to local categorizations, ethnospecies were classified into two groups: ethnospecies that sting (6) including the honeybee *extranjera* (*Apis mellifera*), bumblebees and wasps and ethnospecies that do not sting (9) including stingless bees.

### Ethnospecies known and their uses

**Fig. 4:**
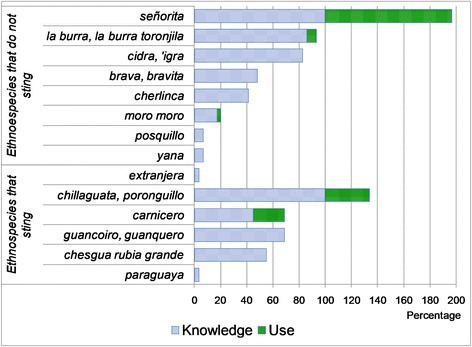
Knowledge and use of melliferous ethnospecies in Baritú surrounding villages

Only 5 of the 15 ethnospecies cited were used by the locals (especially for their honey). At present, the products of *mansita* (*Plebeia* sp. nov.) (also known as *negrillo* and *arropillo* a little further south in the province of Jujuy) are employed by most local people, with citations of at least one type of use by 97% of respondents. To a lesser extent, the present consumption of honey of *señorita* (*Tetragonisca fiebrigi*) (7% of the cites) and of *cherlinca* (*Plebeia catamarcensis*) (3%) was indicated; in both cases, children appear to be the main consumers. Regarding species that sting, the most cited was *extranjera* (*Apis mellifera*) (34% of mentions) followed by *chillaguata* or *poronguillo* (*Polybia ruficeps*) (24%) (Fig. 4).

### Nest collection and management

In the studied area, the *meleo* (local word to refer to the extraction of honey from wild nests) is made exclusively on *mansita* hives. The *meleo* period extends from September to May, being more active in the spring season since, according to local reports, it is the period in which honey is more abundant and has a better flavor. It is recommended not to collect honey when it is cold (between June and August) for two reasons: (1) due to its scarcity of honey in the nest, it is considered better to reserve honey for hive consumption, and (2) because in these period, the honey is crystallized and/or has a sour taste. However, in particular situations—as before the imminence of a childbirth—it is collected independently of the season.

The location of hives and their honey harvesting are complementary activities, that is to say, these tasks are generally carried out during other activities, for example during the search for cattle, dyeing, medicinal plants, or firewood collection and during the preparation of land for sowing, among others. However, if the honey is required, trips to the *monte* are made for that purpose or it is bought from some neighbor. The search for *mansita* is performed on warm sunny days; in this, weather bees are very active which facilitates their detection. During the search, the tree trunk and main branches are observed carefully, trying to notice if there are hollows in the trunk at different heights. If hollows are seen and they are located in the high parts of the tree, locals usually approach the trunk and look up to see against backlight trying to detect flying movements. If the gaps are located in the lower parts, they are examined in detail. Complementarily, the observation of a dark spot —called *holliniado,* due to its resemblance to soot— at the base of holes often reveals the location of a nest. The size of this spot is also considered an indicator of the age of the hive and, therefore, of the amount of honey in it. As it was expressed by one of the interviewees: “the greater the *holliniado*, the older the hive and the greater amount of honey it has”. The interior of the nest is usually elongated with the hive brood disks located in the center and with honey and pollen reserves at both ends. Pollen and honey are stored inside structures technically called pots. Collecting honey is primarily a man’s job, but occasionally is also carried out by women or shared by couples. First, the *colmeneros* or *meleros* (people dedicated to honey harvesting) locate the nest and depending on its location they select the necessary tools (axes, machetes, containers—empty and clean—and water) and proceed to work. If the hive is located in the lower part of the tree, they only dig a hole to extract the products. However, if it is high, the tree is cut down. In the case of ethnospecies that sting, rags or herbs are burnt in order to generate smoke to calm them for collecting the product, similarly to the procedures used in beekeeping.

In most cases, the collected nests are not put in breeding trays afterwards. Sometimes, the nests are returned to the extraction site, to favor the survival of the hives. Although, according to local reports, the collected hives do not usually stay in the same place, but gather the reserves left in the nest and move to another place where the bees build a new nest. This behavior was reported but never actually observed.

During the harvest, once the nest is accessed, the breeding area is set apart from the honey and pollen pots. At the same time, both pot-honey and pot-pollen are separated avoiding mixing them to prevent honey from fermenting. Once separated, the pots with honey are squeezed manually (action of *chumar* locally). The honey is filtered with a clean cloth and stored in the recipient conditioned for this purpose. In each harvested nest, most of what is collected is honey but some of the pollen, wax, and propolis in the nest is also saved. Sometimes, the pots with honey and pollen are transported to the house, where the process is completed. Bags made with bovine (or wild animals’) leather and/or *porongos* (fruits of *Lagenaria siceraria* (Molina) Standl., Cucurbitaceae) were formerly used to transport and conserve honey.

Once extracted and filtered, the honey is placed in fresh places in the houses until it is used or sold. According to the villagers, if honey is extracted in good conditions —that is without being in contact with pollen— it can be kept for more than a year, although generally, the collected honey is consumed over a short time, either pure or in different preparations. The wax obtained is dried in the sun and then cleaned manually by removing impurities, melted and strained. The collected propolis, called locally beehive incense, is also preserved inside the dwellings until its subsequent use.

Regarding nest management, 14% of the informants mentioned that when they harvest the honey, they usually move the portion of the trunks containing the nest towards the periphery of their houses. It was also observed that the nest is transported to clay pots—made locally—which are broken in half and attached and tied with wire or ropes after placing the nest in them. A similar process was described using plastic containers. The use of breeding crates, equivalent to those employed in Meliponiculture enterprises, was occasionally mentioned.

One of Baritú main honey collectors commented that he had built rustic breeding crates with the aim of trying to establish a *meliponario* later, a practice previously tested in the village with *mansita*; in El Lipeo, a family that had rustic crates with *mansita* and *señorita* was also observed. However, there are certain cultural restrictions to the development of this activity. It is considered that by “tying” the nests in breeding crates, the guardian of the animals of the *monte* could generate harm to the rest of the family production.

### Use of the products of *mansita Plebeia* sp. nov. hives

In this study, the more frequently used products of melliferous insects were those coming from *mansita* hives. Regarding other melliferous insects, their use and that of their honeys by ancestors are known but not used at present.

A total of 255 uses were cited, that of honey was the most frequent, with 186 citations, followed by the use of pollen, wax, and propolis, with 29, 27, and 13 citations, respectively. The collected pollen, locally called *flor de colmena* (beehive flower) by Bariteños, is mainly used as food during the *meleo*, or ingested later in the house in its pure state and without additions (22 mentions). The product is also used in the preparation of *anchi* (5 mentions) (maize flour boiled in water, with *naranja agria* (*Citrus aurantium*), honey or sugar) and *guarapo* (alcoholic beverage) (2 mentions). In the first case, the *flor de colmena* is a complementary element that is washed and incorporated into the water before boiling, then it is filtered and discarded and only the water is used in combination with corn flour. In the second case, the obtained beverage, an equivalent to *chicha*, results from the mixture of *flor de colmena*, water, and, to a lesser extent, honey. This preparation is fermented for a week -or less- if room temperatures favor fermentation.

**Fig. 5:**
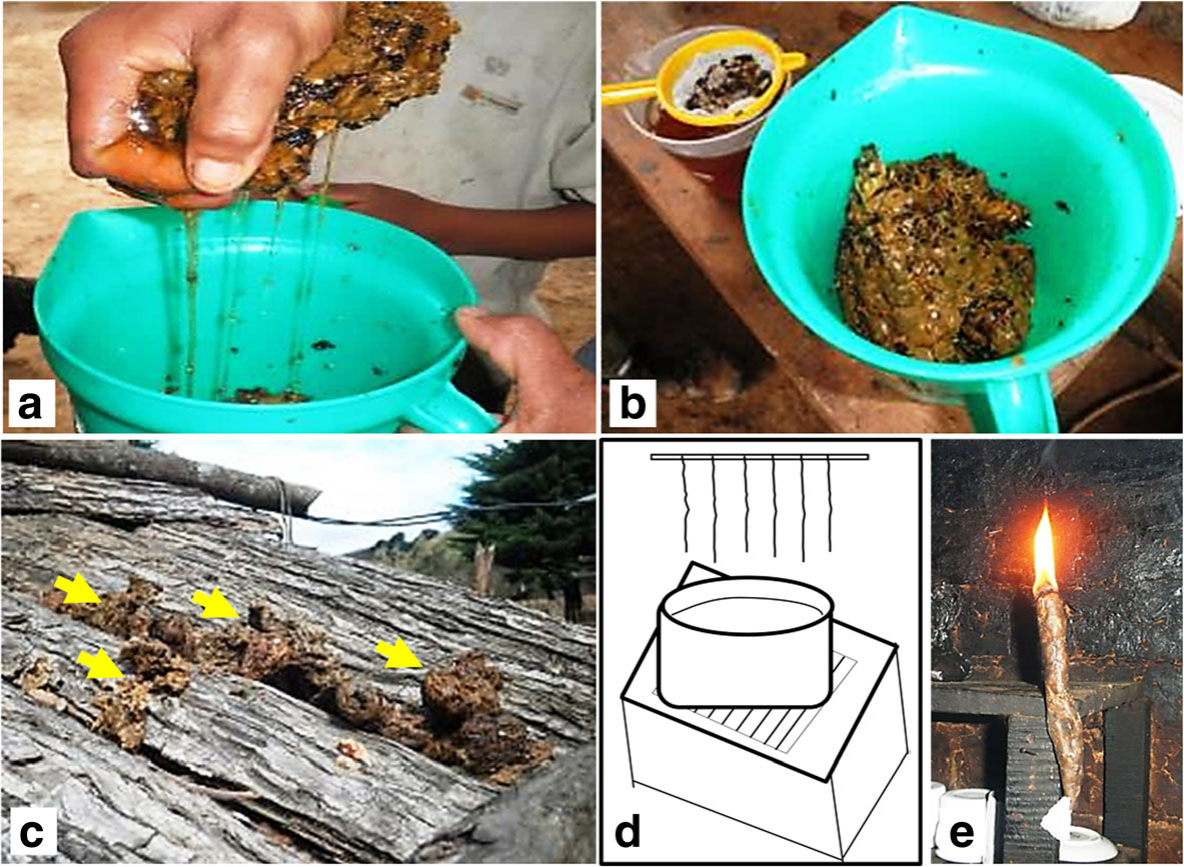
Elaboration of home made candles from wax of *mansita* (*Plebeia* sp. nov.). **a** Manual pressing of honey. **b** Honey filtering and extraction of wax. **c** Sun drying of wax (marked by yellow arrows). **d** Elaboration of homemade candles according to the second procedure. In the illustration, a fastener with cloth wicks to be put into boiled water with a mix of melted wax and fallow. **e** Candle obtained with procedure number one

The wax is used in the production of candles (25 mentions) following two different procedures: (1) The wax is collected by kneading with hands, then softened—placed near the fire—and fractioned. Each fraction is stretched on a smooth surface, a strip of cloth is placed at one end of the mass and rolled up, taking care that the fabric is in the center, like a wick (2). First, the wax is placed in a burlap bag, or other material that allows filtering, and is immersed in hot water. In this way, the wax melts away from the canvas, separating from impurities, and floats on the hot water from where it is withdrawn with a spoon. The same process is followed in another pot with beef tallow. Both clean elements are mixed in equal parts (and put into water). Separately, cloth wicks are prepared and attached by one end to a fastening element (e.g., a twig or rod, wire or iron) leaving the other end free. Subsequently, taking the artifact from the fastener, the wicks are immersed and drawn into the water containing the dissolved mixture (wax and tallow). In this way, the wax adheres to the wick, which quickly solidifies at room temperature. The procedure is repeated several times until the desired candle thickness is reached so this second technique requires more time. Candles with honeybee *extranjera* wax are also made using the same procedure (Fig. 5).

The processes followed for obtaining candles differ in the mode of preparation and their final product. In the first case, the procedure is simple and the product is a dark candle (because of the impurities in it), while in the second case, the procedure is more arduous getting light colored candles, free from impurities.

Despite not being a current practice, two people indicated the use of wax for the assembly of animal figures, particularly *llama* (*Lama glama* L.) as a propitiatory ritual to attract rains during extraordinary droughts. Tradition indicates that these figures should be made and placed in high places exposed to the sun (e.g., the roof of houses). Direct contact with sunlight and “dripping” of melted wax evokes storms.

In relation to propolis, locally called *incensio*, *incienso*, or *liga,* they are used as a fuel element when starting the fire and for cultural purposes to smoke in the *limpias* (ritual healing process, performed by a healer in order to counteract the harmful effects of some cultural ailments, i.e., *susto, aire*, or supernatural ailments, i.e., *aicadura, agarradura*). In turn, it is also used to “ward off storms” for which it is burned—alone or with candles. They are arranged in high sectors or the periphery of the dwelling so that they smoke for a while and favor the “dismantling” of the storms.

Regarding honey, it is used as a dietary supplement (76 mentions), followed by its use for medicinal purposes (71), for sale or barter (26 mentions), and in cultural matters (in ritual or religious offerings) (13 mentions). Regarding the mode of use (both in food and medicine), there is a preponderance for its incorporation in infusions (58%), followed by uncooked mixtures (21%), ingested in pure state (13%), and in dilutions or as a complement (4% in both cases).

Table 2 shows the categories we established according to the uses surveyed in the interviews. In the extracting season, the use of honey as food is the main motivation for its search. In fact, it is a highly valued resource, practically a regional delicacy. During the rest of the year, its use is restricted exclusively to the promotion of health (especially in reproductive medicine and the treatment of cultural ailments).

**Table 2:** Uses of honeys of *Plebeia* sp. nov. —*mansita*—in the locality of Baritú

Category		Mode of application	Accompanying elements	Observations	No. of mentions
Food		Pure	–	Generally, the honey is supplied in fast by the morning, but it can be eat also at other times of the day.	14
		Complement	Bread, *mote*—grains of corn in shell boiled, *chicha*—ferment drink—of *Zea mays (maiz)*.	1) At breakfast or as snack. 2) In the elaboration of the *chicha* drink, as a substitute of ground sugar or *chancaca* (*caña de azúcar*, *Saccharum officinarum* unrefined).	17
		Drink (*Aguamiel*)	Water.	The mode of preparation consists in mixing boiled water (the opposite of cold or raw water, as locally called unboiled water), tempered or cold with honey, usually in 3:1 proportions respectively, or sometimes in 2:1 or 1:1.	28
		Sweetener in infusions	Plants: *Camellia sinensis (te)*, *Ilex paraguarensis (mate).*	–	17
Cultural		Offering in honor to *Pachamama*	–	The ceremony of the *Pachamama* (mother earth) is an activity practiced ancestrally by social groups of the Andean region, consisting of offering various foods and beverages to mother earth. When honey is available, it is offered along with the other foods.	1
		Offering in honor to *Los Fieles Difuntos* (Day of deceased faithfuls, day of all souls).	–	In commemoration of the day of the deceased faithful or day of all souls, people are accustomed to making bread with animal shapes and objects (horses, dogs, staircases), sweet and savory foods, and beverages for the departed. When honey is available, it is part of offerings. The set of products is offered to the deceased on November 1st until the conclusion of the following day. After this period, the offerings are lifted by relatives and neighbors.	12
Commercial		Honeys sale	–	For the benefits it grants, the honey is sold to residents of neighboring towns. The product is ordered or purchased in advance to the residents of Baritú.	26
Medicinal	Digestive system	Constipation*	–	The only mention registered requires the use of honey in the pure state (one or two tablespoons of honey).	1
		Diarrhea	Water. Plants: starch of *Zea mays (maiz)*, *Origanum vulgare (orégano)*, *Lepidium didymum (quimpy)*	The honey is incorporated mixed with the starch, or in infusions of *orégano* o *quimpy*	2
		Stomach ache	Water. Plants: *Clinopodium gilliesii* or *C. bolivianum (muña)*, *Lepidium didymum (quimpy), Origanum vulgare (orégano), Tripodanthus acutifolius (c’orpo)*.	Honey is incorporated only with raw water or in infusions of flowers of *c’orpo*, leaves of *orégano* or portions of plants of *muña* or *quimpy*	8
	Respiratory system	Sore throat*	–	For the relief of sore throat, only pure honey is used.	5
		Influenza	Water. Plants: *Citrus limon (limón)*.	The mode of incorporation consists in to mix boiled water together with lemon juice and honey.	6
		Cold	Water. Plants: *Citrus limon, (limón)*, *Anthemis cotula, Matricaria chamomilla, (manzanilla)*.	The mode of incorporation is through infusions of the *manzanilla* for a short time. Later the *limón* juice and honey are added.	19
	Reproductive system	Post partum	Water. Plants: *Adiantum lorentzii (culandrillo),* *Artemisia absinthium, Tanacetum parthenium (ajenco), Clinopodium gilliesii y/o C. bolivianum (muña), Lepidium didymum (quimpy), Melissa officinalis, Minthostachys mollis (toronjil), Origanum vulgare (oregano), Tripodanthus acutifolius (c’orpo).*	The honey is incorporated in infusions of *ajenco, culandrillo, toronjil*, flowers of *c’orpo, quimpy*, *orégano* or *muña.*	30
Total of mentions					**186**

*System or disease not registered treated with honeys in previous studies

In relation to its use as food, the honey of *mansita* is preferred—whether with bread or as a sweetener—mixed with the *mote* or in *chicha* or other refreshing beverages. This honey is considered more digestible than that of the *extranjera* because the latter is *muy cálida* (very warm) and can cause digestive ailments and/or a hot/cold imbalance when consumed in excess.

As a medicine, honey appears to be more important in the treatment of diseases of three body systems: digestive (11 mentions, for diarrhea, constipation, and stomach pain), respiratory (30 mentions use for sore throat, cold, and flu) and reproductive (30 mentions as a depurative for postpartum use) (Table 2). In relation to the role of honey in these mixtures, it is incorporated for medicinal purposes in all ailments associated with hot/cold medicine, sometimes serving nutraceutical purposes and to a lesser extent, in the treatment of digestive ailments where many of the used herbs are considered very bitter.

Medicinal preparations involving honey often have a herbal base (made up of one or more species) (Table 2). There were 83 preparations, involving 11 ethnospecies belonging to 17 species, 14 genera, and 9 families. The botanical ethnospecies that received the largest number of citations were *oregano* (16 mentions), *c'orpo* (9), and lemon (7). The rest of the species were sporadically cited by the interviewees, with one or two mentions.

## Discussion

### Useful melliferous insects

The presence and distribution of *Plebeia. catamarcensis* and *P. droryana* Friese and *P. molesta* (Puls) are noted for the first time to occur in the Northwest of Argentina [49], e.g., sectors of Jujuy (El Fuerte village, Santa Bárbara department and San Lucas village, Valle Grande department). The genus *Plebeia*, e.g., *P. catamarcensis*, may exist also in Salta (Baritú). A new species (*Plebeia* sp. nov., *mansita*), whose distribution extends to the southern sectors of Jujuy yungas (San Lucas village, Valle Grande department), will be described (information provided by Dr. Álvarez of the Museo de La Plata).

In the case of *Scaptotrigona jujuyensis*, its distribution in the Neotropical region is limited to Argentina and to the province of Jujuy according to Camargo and Pedro [47] or to the whole Argentinean northwest according to Roig-Alsina et al. [24]. The latter indicate that the species is denominated *yana* in the northwest and extends its distribution to sites in the Argentine Chaco, where it is registered with the local name of *negrito* or *tapezuá*. Its presence in the province of Salta and within the province of Jujuy is mentioned for the first time. Distribution is supposed in other sectors of the Yungas; it was registered in San Francisco village (Department of Valle Grande, Jujuy) where it is known by the name of *negrillo*. With respect to *Tetragonisca fiebrigi* before its recorded presence for Jujuy and Salta, it was registered for the provinces of Misiones and Tucumán [47].

Most of the citations of the 15 known melliferous ethnospecies refer to the same ethnospecies (*Plebeia* sp. nov., *mansita*). This differs from that recorded in sectors of the Argentine Chaco where the knowledge and use (of honey, pollen and larvae) of 18 ethnospecies for various purposes have been reported [20], and from the Atlantic Forest, with the knowledge of 12 ethnospecies and the use of 9 used of them, emphasizing in the case of the stingless bee *yateí* (*Tetragonisca fiebrigi*) and of the honeybee (*Apis mellifera*) [11, 18].

### Access to the mellifurous resource, collection, and management

Honey harvesting concentration during spring coincides with that observed in other regions. In Misiones, for example, the Mby’a Guarani relate the activities of searching and collecting honeys to those of sowing and harvesting agricultural products in the spring-summer period. Honey collection in winter is avoided because some honeys acquire a sour and bitter taste in this season [17]. Similarly, among the native people of the Argentine Chaco, honey harvesting is subject to seasonal changes and periods of rain or drought, occurring from pre-spring to the end of the wet period in autumn [22]. This contrasts with that observed 50 years ago in the Chaco of Santiago del Estero province where the period between April and June was considered the best for the extraction of honey [21].

The way of looking for hives described in the region is similar to that registered for the creoles inhabiting the north of the province of Misiones [11] where local knowledge recommends to inspect some species of trees during the search, in particular (*Holocalyx balansae* Micheli, Leguminosae, and *Cordia trichotoma* (Vell.) Arráb., Steud., Boraginaceae). On the other hand, Arenas [20] recorded methods followed by chaquenian people not observed in our region, mentioning that hives are usually traced following bees from water sources. The use of *melero* dogs or observation of the behavior of wild animals that consume honey were registered also by Arenas [20] and Halcroft et al. [50] in Australia. These authors mention that the hives are usually located by following worker bees from water sources as well as by attaching threads, hair, or grass to the abdominal segments of the workers for their subsequent pursuit to the hives [50].

In relation to the mode of extraction from the nests, cutting of the trees where the hives are located is also frequently referred to in other regions, as well as the division of gender for these tasks [11, 17, 20]. The transport and preservation of honey (in bags of bovine or wild animal leather, and in fruits of *Lagenaria siceraria*) was also recorded in Misiones [11] among the ethnic groups of Chaco region where bags made from rhea or *suri* (*Rhea americana* L.), as well as from *tapir* (*Tapirus terrestris* L.), *corzuela* or deer (*Mazama americana* Erxlebe) (*Myrmecophaga tridactyla* L.), *quimelero* (*Catagonus wagneri* Rusconi), and *vizcacha* (*Lagostomus maximus* Desmarest), are used [20, 22].

Finally, the breeding of stingless bees in the area is not a frequent activity, although practices similar to those mentioned for other regions have been observed, e.g., the transfer of host logs to house proximity or the use of containers of diverse nature (pottery, plastic) to carry the product [10, 14, 19, 50] was observed. There was no formal breeding of any of the local species, a practice that has been growing in other regions of the country, such as the breeding of *Tetragonisca fiebrigi* in different sectors of Misiones and Chaco [11, 17, 20], *T. angustula* in the locality of Los Naranjos, Province of Salta [51, 52], and *Scaptotrigona jujuyensis* in the province of Formosa [53].

### Use assigned to products from wild bees

The uses registered in the present study coincide, in general terms, with those observed in the same locality, and in nearby sites, during previous ethnobotanical studies [25]. However, they show some differences, such as a markedly lower register of plant species mentioned in medicinal preparations and a greater importance given to the edible use of honey. Its role in the summer economy of the collecting families was also registered in the first period of study. Honey was an important element of exchange with settlers from other sectors, particularly those “of the hill” [38]. These changes may have resulted from different causes; firstly, they could reflect the interest of the person who conducted the interviews in each case (i.e., in this study, we found more details on the importance of honey while Hilgert focused mainly on useful plants) or may derive from different criteria of selection of the interviewees whose knowledge may have differed according to their role in the family.

In itself, *mansita* honey can be considered a functional food, added in its pure state or combined with other natural elements, as observed in other regions, e.g., in the municipality of Nocupétaro (Mexico), where honey is consumed in its pure state or sometimes accompanied with a hot drink [54], in the Toba and Wichi communities of the Argentine Chaco [20, 23], and in the communities in the Atlantic Forest [11, 17] where bees and wasps acquire relevance providing honey, larvae, and pollen for food. Among the exceptions to this rule, mention can be made of that recorded in the interior of the Brazilian Amazon where melipone honey is used only as a medicinal resource and not as a functional food or food supplement [2].

The fact that native honeys are considered of better quality and/or better digestibility than that of *extranjera* honeybee (*Apis mellifera*) agrees with the assessment these resources were given in other regions of Argentina, among ethnic groups of Chaco [20] and Creoles inhabiting the Atlantic Forest [11]. In different local groups, it is common to use honey as a sweetener, although the abandonment of this practice [38] was observed in the region in studies carried out at the end of the last century. We noticed that presently wild honeys are used as food and as a sweetener almost exclusively in the summer season when their collection takes place. This fact has probably gone unnoticed in previous studies.

The records obtained on beverages made from honey, *aguamiel* and maize *chicha* are the first for the region. Given the importance of the use of honey in this type of preparations in the past and all over the world, its use is likely to have been a common practice in the area of study, as well as the existence of other preparations not mentioned here. According to Pastor [55], there are records of this type of preparations dating from 10,000 years ago. For example, in Europe, the mead, an alcoholic beverage, was consumed abundantly by the Greeks, Celts, Saxons, and Germanic tribes of the north [56]. The *aguamiel* prepared in the region is similar to that recorded in the Chaco by Arenas [20] and Medrano and Rosso [23], who use the name of *hidromiel* or *fresh water* to refer to it.

Arenas [20] registered the elaboration of *guarapo* with different honeys but without pollen among the chaqueños. He registered the use of honeys of wasps called *lechiguana* (*Bachygastra lecheguana* Latreille), of *extranjera* honeybee (*Apis mellifera*) and of two stingless bees, *yana* (*Scaptotrigona jujuyensis*) and *pusquillo* (*Plebeia catamarcensis*). It is interesting to mention that the term *guarapo* is not typical for the locality and region of study, being used elsewhere to name other beverages. For example, in the Canary Islands, it refers to the drink obtained from the sap of the Canary palm (*Phoenix canariensis* Chabaud, Arecaceae) from which palm honey, palm wine, or brandy are made [57].

Previous studies show that, in different ethnicities of the American continent, these preparations are of considerable symbolic value and/or are used for medicinal purposes. For example, the Mayans made *balché,* a culturally important drink used to date for ceremonial and curative purposes. It is made by fermenting the bark of *balché* (*Lonchocarpus longistylus* Pittier, Leguminosae) and honey of *Melipona beecheii* in water (at present if these honeys are not available, they can be replaced by honey from *Apis mellifera* or by sugar) [58]. The Aztecs regularly used honey to sweeten and flavor the drink of the gods, *pulque*, and one of the most appreciated drinks today, *chocolate* [15]. In Peru, beverages containing honey from stingless bees are varied and used for different purposes among are those referred to as aphrodisiac drinks and liqueurs based on aguardiente, honey and macerated plants, such as *abuta* (*Curarea tecunarum* Barneby & Krukoff, Menispermaceae), *clavo huascal* (*Tintanus bustoniaceae*), *chuchuhuasi* (*Maytenus macrocarpa* (Ruiz & Pav.) Briq., Celastraceae), *huito* (*Genipa americana* L., Rubiaceae), and *palo de rosa* (*Aniba rosaeodora* Ducke, Lauraceae), among others [59]. One of the well-known and commonly used aphrodisiac beverages is the *siete raíces* liqueur made from barks of seven species macerated in brandy for 10 days, filtered and mixed with honey from some local stingless bees [60, 61].

We observed the use of the abovementioned honeys in the elaboration of *chicha,* a drink with high ritual and symbolic value [62]. We also noticed the preferential use of two honeys, *incienso* and *flor de colmena*, in ritual cures of ailments of cultural origin [25, 26]. Although it has not yet been reported, it is likely that in the area honeys have—or have had—a symbolic value similar to that recorded by Cruz [63] for other American ethnicities. The importance of the use of honey in cultural contexts, particularly as offers in activities related to the ritual of *Pachamama* (ctonic deity) and in honor of the *Fieles Difuntos* (Faithful departed), can be considered similar to the importance given to *yateí* honey (*Tetragonisca fiebrigi*) among native and indigenous groups of the Atlantic Forest, which, although not ritual, is based in the benefits it entails (e.g., nutritional, medicinal) [11, 17, 64].

The three plants most frequently cited and used in medicinal preparations with which honey is combined are *orégano* (*Origanum vulgare* L.), *c'orpo* (*Tripodanthus acutifolius* (Ruiz & Pav.), and lemon (*Citrus limon* (L.) Osbeck). *Orégano* is regularly used in food as an ingredient or condiment [26, 65] and indicated for therapeutic use in respiratory distress [combined with *tomillo* (*Thymus vulgaris* L., Lamiaceae) to treat cough or for the treatment of inflammation, severe pain, and stomach and intestinal flatulence [66]. In our previous study in the area, *orégano* was registered as an important herb for conditions of the reproductive body system (menstruation, pregnancy and postpartum), sometimes acquiring the role of functional or medicinal food (i.e., it is used in meals during the puerperium) [26].

Regarding *c'orpo*, there are records on the use of infusions of its flowers as abortive, during menstruation and as a postpartum depurative [67]. The latter coincides with that recorded here. Observations made by us indicate the addition of *mansita* honey, not mentioned in others.

In the case of lemon, prepared in the form of uncooked mixtures and in combination with *mansita* honey for diseases of the respiratory system (colds and flu), the vegetable is part of a set of citrus fruits (e.g., oranges (*Citrus sinensis* (L.) Osbeck, Rutaceae) and *limas* (*Citrus aurantifolia* (Christm.) Swingle, Rutaceae)) commonly grown in family farms and present in plantations scattered in the forest, used as food in various preparations [67]. However, despite their nutritional use, fruit juices are used for medicinal purposes alone or accompanied by other elements. In a similar way, Zamudio [11] reported the use of lemon juice in combination with stingless bees honey in sectors of the Atlantic Forest, and Reyes-González et al. [54] registered it in the town of Nocupétaro, Michoacán state (Mexico). In the latter, faced with the lack of lemon juice, Reyes González et al. [54] described how the agave mezcal, ethanol, and the pulp of *Crescentia alata* Kunth (Bignoniaceae) fruit served as substitutes.

Chamomile (*Matricaria chamomilla* L., *Anthemis cotula* L.) was cited for the treatment of colds combined with the honey of *mansita.* However, the uses assigned to this plant are very diverse. For example, in urban contexts, *M. chamomilla* is part of the list of vegetables usually used for medicinal purposes in a wide range of diseases: colds [66], digestive disorders (e.g., diarrhea, flatulences in babies), dermatological diseases (e.g., skin rashes), ophthalmologic (e.g., conjunctivitis), as a tranquilizer and aromatic mixtures, among others [65, 67].

*Muña* (*Clinopodium gilliesii* (Benth.) Kuntze, *C. bolivianum* (Benth.) Kuntze) is widely distributed in the Argentine Yungas, including the study site, and is usually collected by villagers during other activities. Infusions of stems and leaves mixed with honey were indicated to alleviate discomforts of the digestive and reproductive systems, particularly stomach pain and during postpartum. According to the literature reviewed, the plant is employed in several uses [26, 68]. Similarly, to that recorded in this study, it was reported for the treatment of gastrointestinal conditions [67, 68] and postpartum [26], being the only difference its combination with *mansita* honey.

In the case of infusions of *quimpy* (*Lepidium didymum* L.) and *toronjil* (*Melissa officinalis* L., *Minthostachys mollis* (Benth.) Griseb.), they are combined with honey in the treatment of digestive diseases (such as stomach pain and diarrhea) similar to that reported by Hilgert [67] and other uses [26].

According to Chaquenian ethnicities, honey is considered a remedy, simply because bees obtain the raw material (nectar and pollen) from plants that have a reputation as a remedy [20]. In turn, preparing beverages or healing food by combining honey with medicinal plants will probably enhance the desired beneficial effects. In this way, in the future, it would be interesting to delve into the motivations of incorporating honey into preparations in order to determine if the melliferous product acts as a sweetener having no effect on the diseases treated or if honey reinforces the medicinal qualities assigned to the vegetables involved in medicinal preparations.

Regarding pollen, the uses observed locally in both food and beverage processing are frequently cited in the literature. According to previous studies, pollen give benefits against anemia, regulate bowel function, whet the appetite, increase work capacity, lower blood pressure and increase the rate of hemoglobin in the blood [69]. In addition, they can be used as preventive or therapeutic in the treatment of prostate cancer [2]. However, it is mentioned that for the consumption of beehive pollen—regardless of the species—its coloration—which would indicate its conservation status—must be observed to avoid potential intoxication [20, 22, 54]. On the other hand, it is interesting to note that in some regions, the pollen in the nests are not consumed; for example, among groups of settlers from the Brazilian Amazon, pollen are known as bee excrement (bee *puchi*) being discarded during the meleo [2].

## Conclusion

This work is a contribution on the ethnobiology of wild honeys in the Andean populations of the Salta Yungas. The findings highlight the richness of melliferous insects and their importance in food, medicine, and the economy of local families.

Data obtained complement the information available in other ethnobotanical studies in the region. Also, the present study is the first one on stingless bees in the area. A new species of the genus *Plebeia* was registered and it was observed that the known distribution of others has widen. Within the framework of the same research, an article on the pollen content of local honey has been published [70]. At present, the first controlled trials of breeding crates are being carried out with the hope of reducing the cutting of trees associated with the harvest of wild hives and guaranteeing hygiene conditions at the time of harvest. It is to be hoped that this report was able to provide some basic knowledge to serve as a tool in safeguarding resources and in promoting the conservation of the natural environments where these bees thrive. Another very important aspect to be studied is the physicochemical and microbiological evaluation of these honeys as well as the detection of their bioactive principles.
